# Assessing the Utility of 18F-Fluorodeoxyglucose Positron Emission Tomography in the Differential Diagnosis Between Spinal Schwannomas and Meningiomas

**DOI:** 10.7759/cureus.18890

**Published:** 2021-10-19

**Authors:** Hiroya Ono, Gentarou Kumagai, Kanichiro Wada, Atsushi Ono, Toru Asari, Masahiro Aoki, Yasuyuki Ishibashi

**Affiliations:** 1 Department of Orthopedic Surgery, Hirosaki University Graduate School of Medicine, Hirosaki, JPN; 2 Department of Orthopedics, Hirosaki Memorial Hospital, Hirosaki, JPN; 3 Department of Radiation and Oncology, Hirosaki University Graduate School of Medicine, Hirosaki, JPN

**Keywords:** dural tail sign, suvmax, meningioma, schwannoma, 18f-fluorodeoxyglucose-positron emission tomography

## Abstract

Objective

The advantage of 18F-fluorodeoxyglucose-positron emission tomography (FDG-PET) for the differential diagnosis of schwannoma and meningioma remains unclear. The purpose of this study was to compare the maximum standardized uptake value (SUVmax) with computed tomography (CT) and magnetic resonance imaging (MRI) findings and assess its utility in the differential diagnosis of schwannomas and meningiomas.

Methods

This study included 42 patients who underwent surgery and had pathological diagnoses of schwannomas (S group) or meningiomas (M group). Multivariate logistic regression analyses were conducted using meningioma prevalence as the dependent variable, and confounders were selected from those with p-values <0.05, including calcification, dural tail sign, tumor volume, and SUVmax at each spinal level as independent variables.

Results

The SUVmax of the spinal canal type at the level of the cervical vertebrae was significantly higher in the M group (4.6 ± 0.8) than in the S group (2.7 ± 1.4; P = 0.017). Multivariate logistic regression analysis showed that the dural tail sign was significantly associated with differential diagnosis between the S and M groups (odds ratio [OR], 0.851; 95% confidence interval [CI], 0.704-1.031, p<0.001).

Conclusions

The dural tail sign on MRI, but not the SUVmax of FDG-PET, was the most useful for the differential diagnosis between schwannomas and meningiomas.

## Introduction

Spinal schwannomas and meningiomas are intradural extramedullary tumors that are mostly benign. These tumors represent 25%-40% of all spinal tumors [1-3]. Computed tomography (CT) and magnetic resonance imaging (MRI) are widely used for the differential diagnosis. The presence of calcification or the dural tail sign (thickening and enhancement of the peripheral dura adherent to the tumor on contrast-enhanced MRI, resulting in the appearance of a tail) are characteristic findings in meningiomas [4-7]. However, reports suggest that certain cases are difficult to distinguish based on these findings, especially the spinal canal type [7]. Since it is necessary to select appropriate surgical methods according to accurate preoperative distinction [2,8,9], CT and MRI alone may not be sufficient.

Positron emission tomography (PET) is a nuclear medical imaging method that uses radioactive isotopes [10]. It has gained popularity as a diagnostic imaging technique for detecting abnormalities in metabolism at the cellular molecular level [11]. Currently, the most commonly used PET tracer in clinical practice is 18F-fluorodeoxyglucose (FDG), which is recognized as a useful modality for the diagnosis of malignant tumors [12]. Although PET has good quantitative capability and is useful for detecting lesions, its ability for anatomical evaluation is limited [10]. However, in recent years, PET/CT fusion devices, which combine CT images, have become popular, and are useful for anatomical evaluation [13].

In the field of orthopedics, FDG-PET is widely used for the qualitative assessment of metastatic cancer and inflammatory diseases [14-17]. However, its utility in spinal cord tumors has not yet been demonstrated. In addition, there have been no reports demonstrating that the maximum standardized uptake value (SUVmax) on FDG-PET may aid in distinguishing between schwannomas and meningiomas at each spinal level, and between benign and malignant tumors.

The purpose of this study was to compare the SUVmax with CT and MRI findings and assess its utility in the differential diagnosis of schwannomas and meningiomas.

## Materials and methods

Subjects

**Figure 1:**
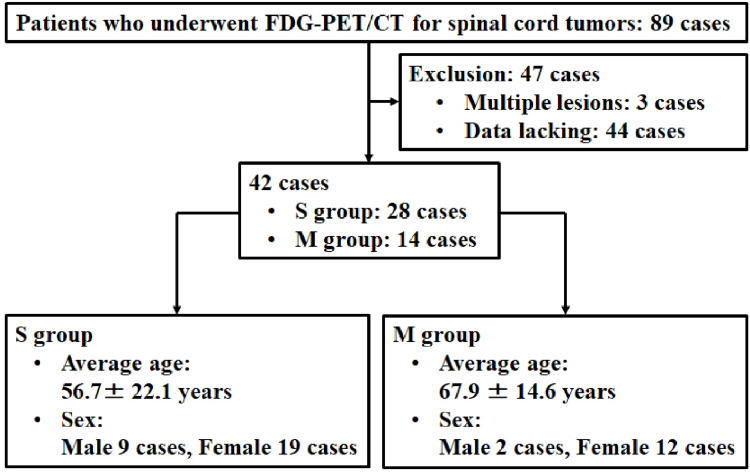
Flow diagram of the study. Among the 89 patients who underwent FDG-PET/CT for spinal cord tumors at our institution between October 2009 and February 2020, 42 patients who underwent surgery and were pathologically diagnosed with schwannomas (S group) or meningiomas (M group) were included in this study. There were 28 and 14 cases in the S and M groups, respectively. The S group included 9 men and 19 women, and the M group included 2 men and 12 women. FDG-PET: 18F-fluorodeoxyglucose-positron emission tomography; CT: computed tomography.

Among the 89 patients who underwent FDG-PET/CT for spinal cord tumors at our institution between October 2009 and February 2020, 42 patients who underwent surgery and were pathologically diagnosed with schwannomas (S group) or meningiomas (M group) were included in this study. Patients with multiple lesions and lack of data were excluded (Figure 1). Informed consent was obtained from all participants in this study. This study was approved by the IRB of the authors’ affiliated institutions (the ethics committee of Hirosaki University Graduate School of​ Medicine, 2019-1038).

Radiographic assessment (CT, MRI, and FDG-PET/CT)

**Figure 2:**
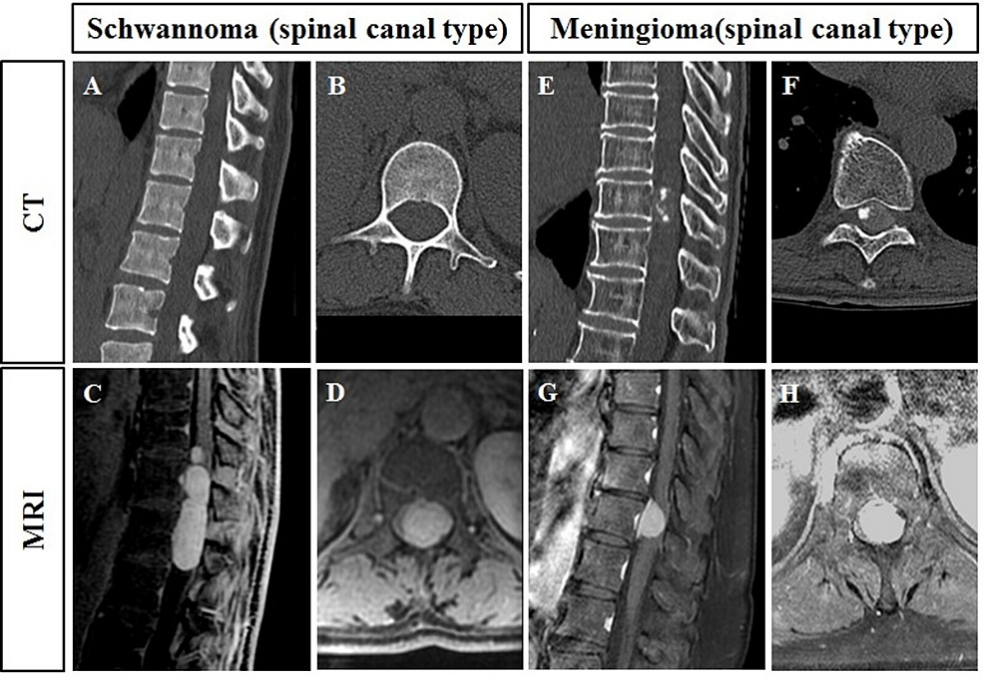
Representative CT and MRI findings of schwannomas and meningiomas. (A–D) Schwannoma (spinal canal type). (E–H) Meningioma (spinal canal type). (A, B, E, F) CT images. (C, D, G, H) Contrast-enhanced MRI findings. CT: computed tomography; MRI: magnetic resonance imaging.

**Figure 3:**
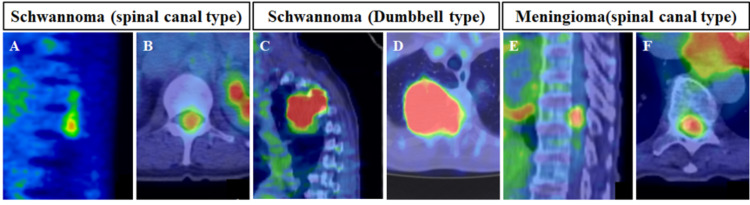
Representative PET/CT images of schwannomas and meningiomas. (A, B) Schwannoma (spinal canal type). (C, D) Schwannoma (dumbbell type). (E, F) Meningioma (spinal canal type). PET: positron emission tomography; CT: computed tomography.

**Figure 4:**
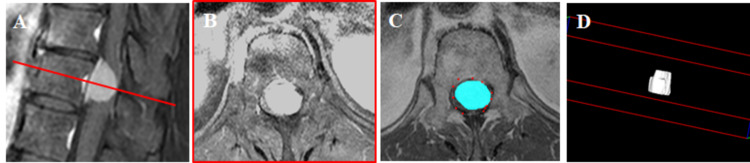
Tumor location and measurement of tumor volume. (A–D) Meningioma (spinal canal type). (A) T2-weighted MR images sagittal plane. (B) T2-weighted MR images axial plane. (C) Contrast-enhanced MR imaging axial plane. (D) The region of the tumor was isolated and a three-dimensional model was created. MR, magnetic resonance.

All patients underwent CT (General Electric Healthcare, Waukesha, WI, USA) (WL: 300, WW: 1200) and contrast-enhanced MRI (Signa HDxt 1.5T, 3.0T, General Electric Healthcare, Waukesha, WI) (TE: 1 ms, TR: 5 ms) of the entire spine, and FDG-PET/CT (General Electric Healthcare, Discovery STE, Tokyo, Japan) before surgery (Figures 2, 3). At our hospital, the CT and MR images were interpreted by orthopedic surgeons, and the FDG-PET/CT images were interpreted by radiologists. The prevalence of calcification in the tumors was evaluated using CT. The tumor level, type (spinal canal type or dumbbell type), location (ventral type or dorsal type), and prevalence of the dural tail sign were evaluated using contrast-enhanced MRI. The tumor volumes in contrast-enhanced MRI were evaluated using MRI reconstruction software (Zed-Edit, Lexi, Tokyo, Japan) (Figure 4) [18]. Two examiners (GK, a spine surgeon with 17 years of experience; and HO, an orthopedic surgeon with 7 years of experience) measured the tumor volume. The intra-rater reliability of this measurement (intraclass coefficient correlation [ICC (1, 1)] by GK was 0.960 (95% confidence interval [CI]: 0.873-0.988; P < 0.001), and HO was 0.956 (95% CI: 0.872-0.976; p < 0.001). Moreover, the inter-rater reliability (ICC (2, 1) by GK and HO was 0.959 (95% CI: 0.849-0.990; P < 0.001). The SUVmax was evaluated using FDG-PET/CT (SUV = specific radioactivity [Bq/g] / injected dose [Bq] / body weight [kg]).

Statistical analysis

The data input and statistical calculations were performed using the SPSS ver.24.0 (SPSS Inc., Chicago, IL, USA) software package. The Mann-Whitney U test was used to test for differences in mean age, body mass index (BMI), duration of disease, tumor volume, and SUVmax between the S and M groups. SUVmax was compared between the two groups at the cervical, thoracic, and lumbar levels for each type of tumor. The chi-squared test was used to test for differences in sex, calcification, dural tail sign, tumor level, tumor type, and tumor location between the S and M groups. Values are expressed as mean ± standard deviation (SD). Statistical significance was set at p < 0.05. To identify independent factors for distinguishing between schwannomas and meningiomas, multivariate logistic regression analysis was conducted. Independent variables with p-values <0.1 in the univariate analysis, including the SUVmax of FDG-PET, were eligible for inclusion in the multivariate models.

## Results

SUVmax on FDG-PET in the S and M groups based on tumor type and spinal level

**Figure 5:**
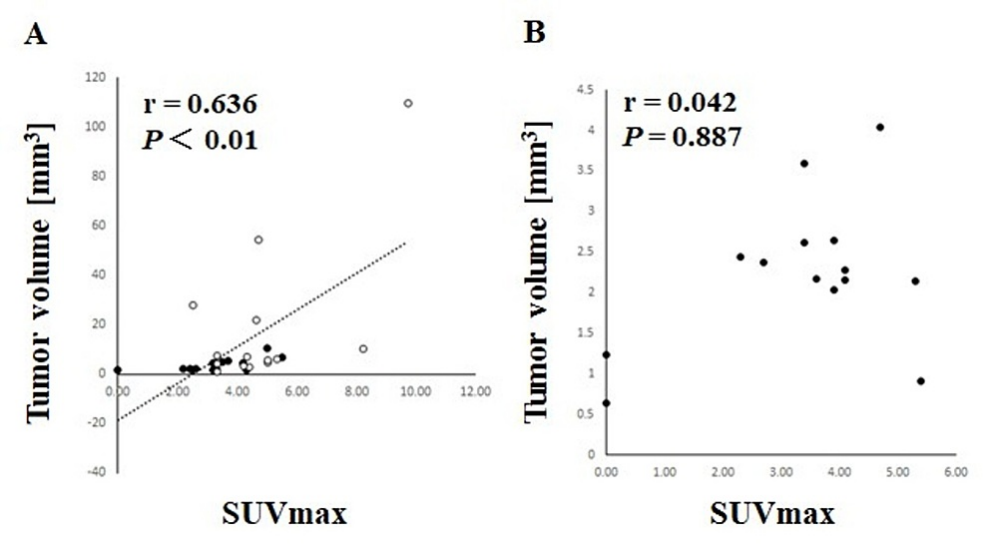
Relationship between SUVmax and tumor volume in the S and M groups. (A) Schwannoma. ○：Dumbbell type, ●：Spinal canal type. (B) Meningioma. ●：Spinal canal type. Significant differences (p < 0.05) were calculated using the *Spearman’s rank correlation coefficient. The black circle indicates the spinal canal type. The white circle indicates the dumbbell type. SUVmax: maximum standardized uptake value.

Although the SUVmax of the spinal canal type tumor at the level of the cervical vertebrae was significantly higher in the M group (4.6 ± 0.8) than that in the S group (2.7 ± 1.4) (p = 0.017), there were no significant differences between the groups in terms of SUVmax in the spinal canal type tumor at all vertebral levels (S group, 3.1 ± 1.1; M group, 3.3 ± 1.7) and at the thoracic level (S group, 3.5 ± 1.0, M group, 2.7 ± 1.6) (Table 1). There was a positive correlation between the SUVmax and tumor volume in the S group (r = 0.636, p < 0.01) (Figure 5). However, there were no correlations between the SUVmax and tumor volume in the M group (Figure 5).

**Table 1:** SUVmax on FDG-PET in the S and M groups based on tumor type and spinal level. ^a^Mean ± SD. ^b^Significant differences (p < 0.05) between the S and M groups were calculated using the *Mann–Whitney U test. SUVmax: maximum standardized uptake value; FDG-PET: 18F-fluorodeoxyglucose-positron emission tomography; S and M group: schwannomas and meningiomas group.

		SUVmax		
		S group (n = 28)	M group (n = 14)	p-value^b^
Spinal canal type				
Level of tumor	All^a^ (n)	3.1 ± 1.1 (15)	3.3 ± 1.7 (14)	0.270
	Cervical^a^ (n)	2.7 ± 1.4 (6)	4.6 ± 0.8 (5)	0.017*
	Thoracic^a^ (n)	3.5 ± 1.0 (7)	2.7 ± 1.6 (9)	0.408
	Lumbar^a^ (n)	2.9 ± 0.5 (3)	- (0)	-
Dumbbell type				
Level of tumor	All^a^ (n)	5.1 ± 1.9 (13)	- (0)	-
	Cervical^a^ (n)	4.9 ± 1.9 (6)	- (0)	-
	Thoracic^a^ (n)	7.6 ± 3.0 (2)	- (0)	-
	Lumbar^a^ (n)	4.4 ± 0.7 (5)	- (0)	-

Univariate and multivariate predictors of differential diagnosis between the S and M groups

Table 2 summarizes the univariate and multivariate predictors of the differential diagnosis between the S and M groups. The mean age of the M group was significantly higher than that of the S group (p = 0.017). There were no significant differences in sex, BMI, or duration of disease between the S and M groups. The prevalence of tumors at the level of the lumbar vertebrae was significantly higher in the S group than in the M group (p = 0.044), and the prevalence of tumors at the level of the thoracic vertebrae was significantly higher in the M group than in the S group (p = 0.047). However, there were no significant differences between the S and M groups in terms of the prevalence of tumors at the cervical level. In terms of the type of tumor, the prevalence of the spinal canal type was significantly higher in the M group (p < 0.001), while that of the dumbbell type was significantly higher in the S group (p < 0.001). Regarding tumor location, the prevalence of the ventral type was significantly higher in the M group (p < 0.001), while that of the dorsal type was significantly higher in the S group (p < 0.001). The prevalence of calcification was significantly higher in the M group than that in the S group (p < 0.001). The prevalence of the dural tail sign was also significantly higher in the M group than that in the S group (p < 0.001). The tumor volume was significantly higher in the S group (11.5 ± 22.1 cm^3^) than in the M group (2.2 ± 0.9 cm^3^) (p = 0.004).

**Table 2:** Univariate and multivariate predictors of differential diagnosis between S and M groups. OR: odds ratio; BMI: body mass index; CI: confidence interval; SUVmax: maximum standardized uptake value. ^a^Mean ± SD. ^b^Significant differences (P < 0.05) between the S and M groups were calculated using the *Mann–Whitney U test and the ♰ chi-square test. ^c^Significant differences (P < 0.001) between the S and M groups were calculated using #multivariate logistic regression analysis.

Variables	S group (n = 28)	M group (n = 14)	p-value^b^	Multivariate analysis		
				OR	95% CI	p-value^c^
Age^a^ [years]	56.7 ± 15.1	67.9 ± 14.6	0.017*	0.080	-0.001-10.006	0.165
Male sex [%] (n)	32.1 (9)	16.7 (2)	0.195	0.065	-0.056-0.195	0.266
BMI^a ^[kg/m^2^]	22.0 ± 4.0	22.9 ± 2.6	0.107	0.043	-0.009-0.021	0.447
Duration of disease^a ^[months]	11.8 ± 10.5	10.0 ± 7.0	0.842	-	-	-
Cervical level of tumor [%] (n)	71.4 (12)	35.7 (5)	0.747	-	-	-
Spinal canal type of tumor [%] (n)	46.4 (13)	0 (0)	0.002^♰^	0.010	-0.147-0.168	0.894
Ventral location of tumor [%] (n)	25.0 (7)	85.7 (12)	<0.001^♰^	--0.095	-0.231-0.051	0.204
Calcification [%] (n)	0 (0)	42.9 (6)	<0.001^♰^	-0.009	-0.196-0.172	0.896
Dural tail sign [%] (n)	0 (0)	92.9 (13)	<0.001^♰^	0.851	0.704-1.031	<0.001^#^
Tumor volume^a ^[cm^3^]	11.5 ± 22.1	2.2 ± 0.9	0.004*	-0.009	-0.004-0.003	0.894
SUVmax	4.1 ± 1.8	3.3 ± 1.7	0.743	-0.42	-0.048-0.026	0.538

Multivariate logistic regression analysis showed that the dural tail sign was significantly associated with differential diagnosis between the S and M groups (odds ratio [OR], 0.851; 95% CI, 0.704-1.031, p < 0.001).

## Discussion

To the best of our knowledge, this is the first study to investigate whether SUVmax on FDG-PET is useful for the differential diagnosis between schwannomas and meningiomas. Although the SUVmax of the spinal canal type tumor at the level of the cervical vertebrae was significantly higher in meningiomas than in schwannomas, the dural tail sign on MRI was the most useful for the differential diagnosis between schwannomas and meningiomas. This finding was similar to the results of previous reports.

Dural resection and reconstruction are required in cases of intradural meningiomas; therefore, it is crucial to distinguish between schwannomas and meningiomas before surgery [19]. In this study, both tumors were differentiated by intraoperative pathological examinations in all cases. However, this is not possible at all institutions. Therefore, the dural tail sign, and not FDG-PET, was the most useful finding for the differential diagnosis between schwannomas and meningiomas in this study.

In this study, the age of the M group was significantly higher than that of the S group. Previous studies have reported that schwannomas occur most frequently in patients in their thirties and forties [20-22], and meningiomas are most common in patients in their fifties, sixties, and seventies [19,23]; these findings are in concordance with the results of the present study. Previous studies have reported that meningiomas are more common in women [24,25]. In our study, although not significant, patients with meningiomas tended to have more complications than those with schwannomas.

The present study demonstrated that the prevalence of tumors at the level of the thoracic and lumbar vertebrae was significantly higher in the S group than in the M group. De Verdelhan et al. [4] reported that meningiomas are most common at the level of the thoracic vertebrae, but rarely occur at the level of the lumbar vertebrae. The results of this study agree with those of the previous studies. In this cohort, dumbbell-type tumors were found only among schwannomas; reports have also suggested that schwannomas account for the majority of dumbbell tumors [26]. In terms of tumor location, the prevalence of the ventral type was significantly higher in the M group than in the S group (p < 0.001). Reports suggest that meningiomas are mostly located anterior to the spinal cord [23], which concurs with our findings. We found that the prevalence of calcification was significantly higher in the M group than in the S group. The reported prevalence of calcification in meningiomas is as low as 2%-5% [23]. However, in cases where calcification is observed, the tumor is usually a meningioma; this characteristic is useful in the differential diagnosis of schwannomas [7]. In this cohort, the prevalence of the dural tail sign was significantly higher in the M group. On contrast-enhanced MRI, this sign has been reported to be a characteristic finding in meningiomas [4-6,23]. Therefore, as previously reported, the presence of calcification and the dural tail sign on CT and MRI were both considered useful for distinguishing between these tumors.

The present study demonstrated that in spinal canal-type tumors at the level of the cervical vertebrae, the SUVmax was significantly higher in the M group than in the S group. However, there were no significant differences between the groups in terms of SUVmax after correction of tumor volume in the spinal canal type at all vertebral levels, including the cervical and thoracic levels. Do et al. [27] described the pattern of 18F-FDG uptake in the spinal cord in 92 patients with non-central nervous system malignancies. They defined the cord-to-background ratio as the ratio of the SUVmax of the entire spinal cord to that of the L5 levels; they found that the mean ratio decreased at each spinal level in a craniocaudal direction. In this study, the SUVmax of spinal cord tumors did not decrease at any spinal level in the craniocaudal direction. Tomura et al. [28] found that in cases of ependymoma, the SUVmax with 11CMetionine (MET) and FDG was 2.2 to 3.5 and 3.5 to 11.2, respectively. Matsumoto et al. [29] reported that the mean SUVmax of the primary malignant spine/spinal tumors was 8.4 ± 6.2. In this study, the SUVmax of spinal schwannomas and meningiomas was higher than that of the normal spinal cord and lower than that of malignant spinal cord tumors. Therefore, in cases of malignant spinal cord tumors, including metastases from cancer, the SUVmax that we found may be considered a reference value. Moreover, we demonstrated a positive correlation between SUVmax and tumor volume in the S group. This may be because the tumor volume of the dumbbell type in the S group was larger than that in the spinal canal type. Although our findings suggest that SUVmax is not useful for the differential diagnosis of these tumors compared with CT and MRI, the evaluation of schwannomas using PET/CT needs tumor size correction because schwannomas are variable in their size. Based on the results of this study, performing PET/CT in all cases due to radiation exposure is not recommended. However, it may be useful for differentiating between tumors in patients with a history of malignant disease or metastasis [17]. It has been reported that the average SUVmax of the metastatic spinal tumors was 6.7, but in this study, the average SUVmax values of the S and M groups were 4.1 and 3.3, respectively. Therefore, the SUVmax on FDG-PET in this study varied when compared with that of malignant tumors.

This study had certain limitations. First, we only included World Health Organization grade I tumors; grade II or III tumors were not included because the number of cases was small. A previous study reported the utility of 18F-FDG-PET in the assessment of tumor grade in intracranial meningiomas [30], including histological grades and types, which may further improve the preoperative diagnosis of these tumors. Second, previous studies considered the pathology and MIB-1 index [28,30], but these parameters were not evaluated in this study. Third, since the number of cases was small, no clear cutoff value of the SUVmax was found in the spinal canal-type tumors at the level of the cervical vertebrae, which would distinguish spinal schwannomas from meningiomas. Fourth, despite a relatively small sample size, the standard deviation of SUVmax values at each level in the spinal canal type was small in this study. In the future, well-designed large-scale studies will be needed to further explore the association between SUVmax and spinal cord tumors. Fifth, we cannot reveal a precise mechanism to state that the SUVmax of the spinal canal type tumor at the level of the cervical vertebrae was significantly higher in the M group than that in the S group. Although there were several limitations, future studies, including larger samples, histological grades and types, and molecular characteristics, may further improve the preoperative diagnosis of these tumors.

## Conclusions

We investigated whether SUVmax on FDG-PET is useful for the differential diagnosis between schwannomas and meningiomas. Although the SUVmax of the spinal canal type tumors at the level of the cervical vertebrae was significantly higher in meningiomas than in schwannomas, the dural tail sign on MRI was the most useful for the differential diagnosis between schwannomas and meningiomas.
